# Temperature Gradient-Induced Microstructural Evolution and Wear Resistance Enhancement in High-Manganese Steels by Laser Transformation Hardening

**DOI:** 10.3390/ma19132725

**Published:** 2026-06-25

**Authors:** Shuwen Wang, Kai Liu, Wenting Zhu, Liang Hao

**Affiliations:** 1 College of Mechanical Engineering, Tianjin University of Science and Technology, Tianjin 300457, China; 2 China Road and Bridge Corporation, No. 88C, Andingmenwai Avenue, Beijing 100011, China

**Keywords:** high-manganese steel, laser transformation hardening, microhardness, impact toughness, wear resistance

## Abstract

Despite its excellent impact toughness and work-hardening capacity, high-manganese steel (HMS) suffers from low initial hardness, limiting its wear resistance under low-stress conditions. Conventional surface hardening methods for HMS involve high cost and intensive energy consumption and produce only shallow hardened layers; moreover, the understanding of laser transformation hardening in HMS remains insufficient. To address these gaps, this study employs a high-energy-density laser for rapid and precise surface modification of Mn13 HMS. The studied Mn13 steel contains 1.98 wt.% Cr, which contributes to solid-solution strengthening and influences the phase transformation behavior during laser transformation hardening. By optimizing the laser power, a well-defined laser-quenched layer with a gradient microstructure along the thickness direction is obtained. Microhardness at the surface treated by laser transformation hardening at 1.5 kW improved significantly, primarily due to grain refinement and a dense dislocation network. The small fraction of martensite contributes indirectly by generating geometrically necessary dislocations and acting as local barriers to dislocation glide. Along the depth direction, the microhardness varies with the gradient microstructure: coarse columnar grains at intermediate depths cause a slight decrease in microhardness, while the substrate restores it. Correspondingly, the laser-quenched surface exhibits improved wear resistance, as indicated by reduced friction coefficient, wear depth, and wear volume, and the dominant wear mechanism shifts from adhesive to abrasive wear. Importantly, this gradient configuration maintains a mechanically compatible transition between the quenched layer and the substrate, preserving impact toughness comparable to that of the untreated material.

## 1. Introduction

High-manganese steel (HMS) is recognized for its excellent wear resistance and low-temperature toughness. As a representative grade, Mn13 steel exhibits an initial hardness of merely 200–220 HB. When subjected to high-intensity impact loading, a significant surface hardening effect can be achieved, accompanied by an impact toughness of ≥60 J [1,2,3,4,5]. Consequently, it is suitable for application in severe wear and impact environments such as mining and crushing machinery (hammer plates or liners) [6,7,8,9]. Its performance becomes less satisfactory under low-stress conditions, where insufficient work hardening leads to poor wear resistance, resulting in rapid material loss and shortened service life. Even under high-stress conditions, the inherently low initial surface hardness can still limit its service life during the early stages of operation [10].

Currently, alloying and heat treatment represent the two primary technical routes for enhancing the hardness of high-manganese steel. Li et al. [11] reported that incorporating 3–5 wt.% Cr can effectively improve the hardness and strength of the alloy through solid-solution and precipitation strengthening. Excessive Cr led to carbide coarsening, which, in turn, reduced toughness. In a systematic study, Zellagui et al. [12] indicated that solution treatment at 1050 °C for 2 h followed by water quenching is optimal, as it produces a fully austenitic microstructure and consequently improves both hardness and wear resistance. Although these methods have been widely adopted in engineering practice, certain limitations persist. Alloying typically involves costly or specialized elements and increases material cost and process complexity. Regarding conventional heat treatment, the primary limiting mechanism is not merely energy consumption, but rather the stabilization of austenite and the overall chemical balance. Specifically, because high-manganese steel possesses a stable austenitic microstructure, improper heat treatment parameters (e.g., excessive temperature or insufficient cooling rate) can disrupt the chemical balance, leading to carbide precipitation or austenite decomposition, which in turn degrades impact toughness [13]. In addition, conventional heat treatment still requires prolonged high-temperature heating and controlled cooling, inevitably increasing energy consumption and carbon emissions. Consequently, both approaches are constrained by metallurgical factors and environmental sustainability and cannot fully meet the requirements of green manufacturing and low-carbon production [14,15].

Under this background, surface strengthening techniques based on process control rather than composition optimization are attracting increasing attention. By generating high-density dislocations, twins and residual compressive stresses, conventional surface pre-hardening methods, including explosive hardening and mechanical impact, enhance the wear and deformation resistance of HMS under low-impact loading and low-stress service conditions [16]. For example, Duong et al. [17] found that explosive treatment forms a hardened surface layer in HMS, markedly improving hardness while minimizing residual macroscopic deformation. Similarly, Ngo et al. [18] demonstrated that shot peening treatment induces strong interactions between twins and dislocations near the surface of HMS, forming a nanocrystalline layer extending several tens of microns and markedly enhancing wear resistance. While shot peening is widely recognized for improving fatigue life without introducing defects [19], its effectiveness in enhancing the wear resistance of HMS under low-stress abrasive conditions is limited. This is because shot peening typically produces only a shallow hardened layer and cannot tailor a gradient microstructure that balances surface hardness with substrate toughness [20,21].

Building on these limitations, laser-assisted processing offers a promising alternative. Among these techniques, laser transformation hardening (LTH) utilizes a high-energy-density laser beam to rapidly heat and cool the material surface, inducing a steep temperature gradient. This rapid thermal cycling enables the formation of a deeper and more uniform hardened layer with a precisely controlled temperature gradient, as well as a gradient microstructure that provides a mechanically compatible transition between the hardened layer and the substrate [22,23]. Consequently, for applications where wear resistance under low-stress abrasive conditions is the primary concern, LTH offers distinct advantages over shot peening, particularly in its ability to simultaneously enhance surface hardness, maintain impact toughness, and avoid sur-face defects. Such rapid heating and self-quenching process induces a steep temperature gradient, leading to martensitic transformation, grain refinement, and dislocation strengthening in the treated layer, thereby significantly increasing surface hardness and wear resistance. Sun et al. [24] performed laser surface processing on an austenitic Hadfield manganese steel (~13 wt.% Mn) and fabricated a ~600 μm-thick gradient nanostructured layer along the depth direction, achieving a substantial increase in surface hardness. Similarly, Liu et al. [25] applied high-power, high-speed laser quenching to 65Mn steel and observed a 42% increase in microhardness in the laser-quenched zone, accompanied by a notable reduction in wear depth. However, due to the highly alloyed composition characteristics and the complex microstructural evolution behavior of HMS, it exhibits significant sensitivity to energy input and thermal cycle parameters under rapid laser heating and cooling conditions. The controllability of microstructure and strengthening effects remains to be improved, resulting in relatively insufficient research and application of laser quenching technology in this material system. A critical gap in previous laser surface treatment studies on HMS is that the exploitation of temperature gradient induced depth dependent microstructures for synergistic enhancement of hardness, toughness, and wear resistance has not been systematically addressed, particularly regarding the formation of an intermediate columnar grain zone and the role of incomplete dynamic recrystallization. The objective of this study is to elucidate how the temperature gradient induced microstructural evolution along the thickness direction governs these mechanical properties. To achieve this, we systematically investigated laser transformation hardening of SCMnH11 HMS under different laser powers at 2 mm/s, characterized the microstructural changes including phase transformation, recrystallization behavior, and dislocation configuration, and correlated them with hardness, impact toughness, and wear resistance. This reveals, for the first time in laser-hardened HMS, a non-monotonic hardness profile arising from a columnar to equiaxed transition, and demonstrates that incomplete recrystallization governs a transition from adhesive to abrasive wear. Importantly, this gradient microstructure preserves impact toughness comparable to untreated material while significantly increasing surface hardness. By establishing these quantitative structure-property correlations, this study moves beyond parameter optimization toward a predictive framework for designing durable surface layers on HMS components.

## 2. Experimental Materials and Methods

### 2.1. Laser Transformation Hardening Treatment

A forged Mn13 steel plate 10 mm thick was used in this present study. The chemical composition of HMS was measured to be (wt.%): 0.91% C, 0.81% Si, 12.51% Mn, 1.98% Cr, and the balance Fe. The chemical composition of the high-manganese steel was determined by inductively coupled plasma optical emission spectrometry (ICP-OES). The spectrometer is manufactured in Teledyne Leeman Labs, Hudson, NH, USA, and its model is Prodigy. Following water toughening treatment, microstructural characterization of the HMS revealed a fully face-centered cubic (FCC) austenite, exhibiting an average grain size of about 90 μm. The microhardness of the surface was determined to be 259.5 HV1.

**Figure 1:**
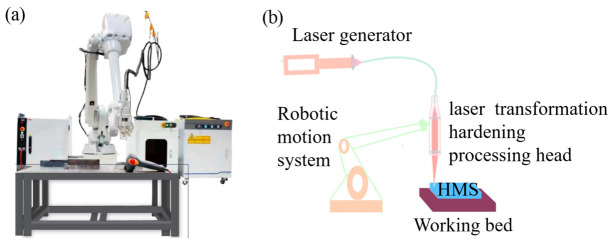
Laser transformation hardening device of HMS surface. (**a**) Experimental setup. (**b**) Schematic diagram of LTH.

As shown in Figure 1, the LTH system consisted of a laser, a laser transformation hardening processing head, a robot arm, and a cooling device. This instrument is manufactured in Taizhou Chuangying Laser Technology Co., Ltd., Taizhou, China. A laser source with a wavelength of approximately 1064 nm and an output power of 6000 W was employed. The laser transformation hardening head connected to this source consists of a collimation unit, a beam homogenizer, and a focusing mirror assembly, which converts the incident Gaussian beam into a rectangular top-hat beam profile. The output spot dimensions are 20 mm × 2 mm, with a uniform energy distribution along the longer axis. The head is mounted on a six-axis robotic arm, allowing precise control of the stand-off distance and incidence angle during processing. During laser transformation hardening, nitrogen gas was used as the cooling medium. The laser was operated in pulsed mode at a frequency of 20 kHz. The defocus distance was set to 400 mm relative to the specimen surface. The HMS was sectioned into rectangular bars (120 mm × 20 mm × 10 mm), which were subsequently secured onto the worktable. Before LTH treatment, the working axis was finely tuned to ensure stable focusing of the laser beam and precise coverage of the intended treatment zone. The experiment was subsequently implemented by irradiation of the specimen surface with the focused laser beam.

Initial experiments aimed at parameter optimization led to the selection of a laser power range from 1 to 3 kW and the scanning speed was 2 mm/s. For clarity and brevity, a simplified nomenclature was adopted, whereby specimens treated at 1, 1.5, 2, 2.5, and 3 kW were labeled as 10HMS, 15HMS, 20HMS, 25HMS, and 30HMS, respectively. The area energy density was calculated as follows:

(1) E = P/(v.w)

where E is the area energy density (J/mm^2^), P is the laser power (W), v is the scanning speed (mm/s), and w is the laser spot width along the scanning direction (mm). Thus, the energy densities for 1.0, 1.5, 2.0, 2.5, and 3.0 kW are 250, 375, 500, 625, and 750 J/mm^2^, respectively. The laser treatment was performed in a single pass without track overlap.

### 2.2. Microstructure and Performance Analysis

Preliminary microstructural observation of the cross-sections of laser-hardened specimens was conducted using an IE500M optical microscope (OM). The instrument is manufactured in Ningbo Sunny Instrument Co., Ltd., Ningbo, China. Subsequently, electron backscatter diffraction (EBSD) analysis was performed on a Gemini 300 system operated at an accelerating voltage of 20 kV. The instrument is manufactured by Zeiss, and its place of origin is Oberkochen, Baden-Württemberg, Germany. Prior to OM and EBSD examinations, all laser-hardened specimens were sequentially ground, mechanically polished, and then chemically etched in a 4% nitric acid alcohol solution for 5 s to eliminate stress effects induced during the preceding preparation steps. Detailed microstructural characterization at the nanoscale was conducted using a high-resolution transmission electron microscope (HRTEM, Talos F200X G2) operated at an accelerating voltage of 200 kV. The electron microscope is manufactured by Thermo Fisher Scientific, and its production site is located in Brno, Czech Republic. The TEM specimens were prepared by first mechanically grinding the specimens to a thickness of approximately 50 μm by silicon carbide abrasive papers, and then punching them into discs 3 mm in diameter. These discs were subsequently electro-polished using a Struers TenuPol twin-jet electropolishing with a solution consisting of 10 vol% perchloric acid and 90% ethanol at a constant temperature of −25 °C. The instrument is manufactured by Struers, and the company is headquartered in Ballerup, Denmark.

A D8 Advanced X-ray diffractometer with Cu Kα radiation was adopted to analyze phase compositions of samples before and after laser transformation hardening. This instrument is made by Bruker in Karlsruhe, Germany. Diffraction signals of retained austenite were acquired within the 2θ interval from 40° to 100°, and the scanning step was set at 2.5°. The volume fraction of retained austenite was calculated via integrated intensity analysis of characteristic diffraction peaks. The selected peaks covered (200) and (211) planes for BCC structure, as well as (200), (220) and (311) planes belonging to FCC structure [26].

(2) $$V_{\mathrm{RA}}=1.4I_{FCC}/(I_{BCC}+1.4I_{FCC})$$

In this context, I_BCC_ and I_FCC_ correspond to the average integrated intensities of the diffraction peaks from the BCC and FCC phases, respectively.

Laser-hardened Depth-gradient microhardness tests were carried out on laser-hardened samples with a Zwick microhardness tester (ZwickRoell ZHVµ). The instrument is manufactured by ZwickRoell, a company headquartered in Ulm, Germany. The indentation load was kept constant at 10 N, and the dwell time was set to 15 s. Five separate indentations were made for each testing position, and the corresponding microhardness value was calculated as their average. The instrumented impact tests aimed at analyzing how laser transformation hardening parameters affect the toughness of HMS material were carried out on a ZBC 2452-C pendulum impact tester fitted with a force-time recording system. The instrument is manufactured by MTS/SANS, specifically by MTS Industrial Systems Co., Ltd. in Shenzhen, China. Impact specimens were prepared according to the standard Charpy V-notch (CVN) method, with dimensions of 55 mm ×10 mm ×10 mm. Three separate impact tests were performed at room temperature for each set of processing parameters, and the fracture morphology was subsequently analyzed in detail using a SU8600 scanning electron microscope (SEM) operated at an accelerating voltage of 5 kV. The instrument is manufactured by Hitachi and is made in Tokyo, Japan.

LTH Wear tests were carried out in reciprocating mode on the surface of HMS specimens before and after LTH, and the friction coefficients were acquired simultaneously using a UMT-TriboLab tribometer. The instrument is manufactured by Bruker, San Jose, CA, USA. A silicon nitride (Si_3_N_4_) ball with a diameter of 5 mm and a microhardness of 1700 HV1 was employed as the counterpart material. Silicon nitride was selected for its superior stability under high-pressure conditions and excellent microhardness, which enables simulation of severe friction conditions and thereby facilitates effective evaluation of the surface strengthening performance of brake discs. A constant load of 100 N, a sliding radius of 5 mm, and a frequency of 5 Hz were applied during the test. Each test lasted for 30 min. The three-dimensional surface morphology of the specimens before and after LTH was analyzed with a Leica DCM8 3D profilometer, followed by observation using a SU8600 SEM to assess wear--induced morphological changes. The Leica DCM8 3D profilometer is manufactured by Leica Microsystems, Wetzlar, Germany.

## 3. Results and Discussion

### 3.1. Microstructural Evolution

**Figure 2:**
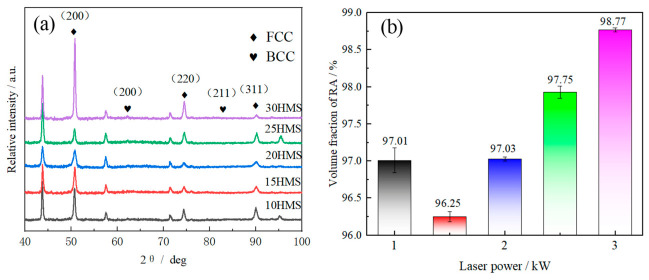
XRD results of the specimens after LTH treatment. (**a**) XRD patterns. (**b**) Evolution of *V*_RA_.

**Figure 3:**
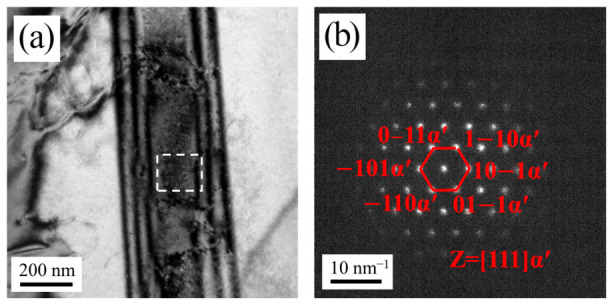
TEM characterization of the α’–martensite phase. (**a**) TEM bright-field image. (**b**) SAED pattern taken from the region enclosed by the white dashed square in (**a**).

LTH The XRD spectra of HMS after LTH are illustrated in Figure 2. Diffraction peaks associated with BCC are detected in all laser-quenched specimens, indicating that martensitic transformation was successfully achieved via laser surface treatment. The V_RA_ was calculated from the integrated intensities of the BCC and FCC diffraction peaks according to the method described by Equation (2). The results showed that the martensite content reached a maximum value of 3.75% at a laser power of 1.5 kW, and a further increase in laser power led to a reduction in martensite content. In high-manganese steels, stress-induced martensite can form as BCC α′ or HCP ε, with possible XRD peak overlap. In this study, the peaks at ∼44.5° and ∼64.8° correspond to BCC α′-martensite, while characteristic HCP ε-martensite peaks (e.g., ~41°, ~44°, ~47°) were not clearly resolved. Under rapid thermal cycling such as laser transformation hardening, the transformation may proceed directly from γ-austenite to α′-martensite without an intermediate HCP phase. Nevertheless, due to possible peak overlap and the small martensite fraction, trace amounts of ε-martensite cannot be completely excluded based on the current XRD data. To provide direct crystallographic evidence and address this concern, we performed additional TEM characterization. As shown in Figure 3a, the lath-like martensite morphology was clearly observed. More importantly, the selected area electron diffraction (SAED) pattern taken from the marked region (Figure 3b) is unambiguously indexed as α’-martensite (Z = [111]) without any detectable ε-martensite reflections. This direct crystallographic evidence confirms that the phase is α’-martensite, effectively ruling out the presence of ε-martensite within the analyzed region.

**Figure 4:**
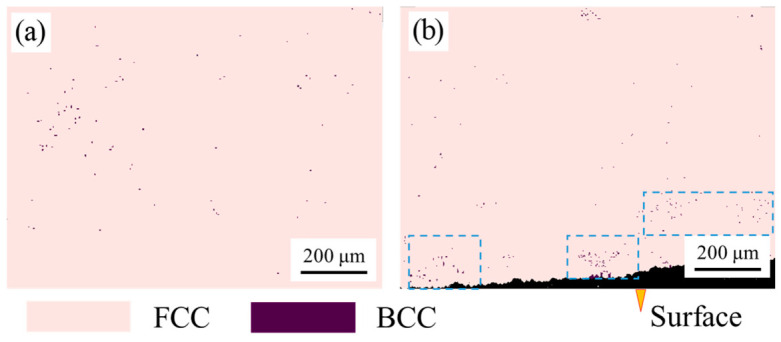
Phase map of 15HMS after LTH treatment. (**a**) Surface. (**b**) Cross-section.

XRD analysis of the 15HMS surface revealed that a significant transformation from austenite to martensite occurred under this power condition. Its surface phase map (Figure 4a) showed that the martensitic phase was uniformly distributed throughout the entire treated region. Examining the cross section of 15HMS (Figure 4b), martensite content decreased gradually from the surface toward the interior, with higher fractions near the specimen surface. This distribution reflected the higher cooling rate in the near-surface region induced by laser irradiation, which promoted martensitic transformation.

**Figure 5:**
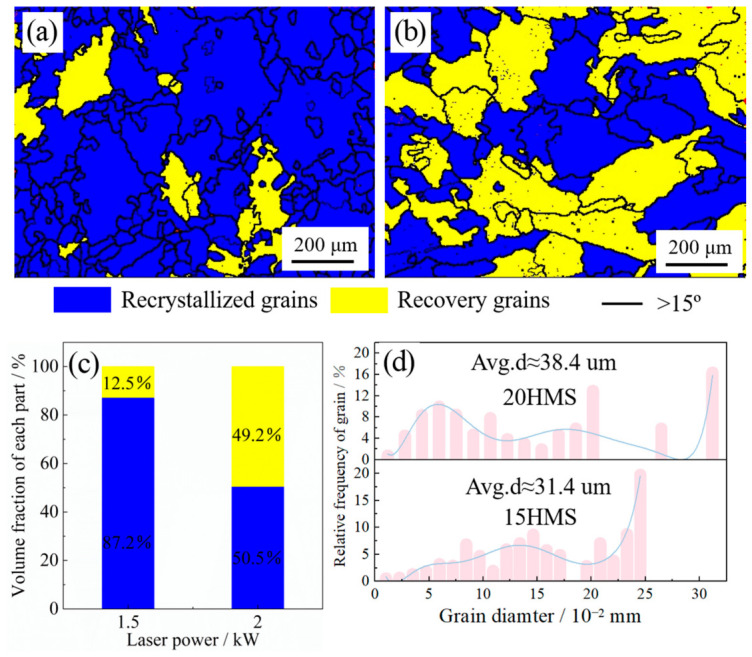
Results of EBSD characterization. (**a**) GOS of 15HMS. (**b**) GOS of 20HMS. (**c**) Proportion of different regions. (**d**) Grain size of 15HMS and 20HMS.

Figure 5a,b show the grain orientation spread (GOS) maps of 15HMS and 20HMS, in which yellow, blue, and red regions correspond to recovered, recrystallized, and deformed grains, respectively [27]. In 15HMS (Figure 5c), recrystallized grains occupied approximately 87.2% of the total area and exhibited a generally finer and more uniform size distribution. By contrast, the recrystallized area fraction in 20HMS is markedly lower, covering about 50.5% of the area. Additionally, the grain size also shows a clear coarsening trend. These microstructural differences were further corroborated by the grain size statistics displayed in Figure 5d. According to the statistical results, the average grain size of 15HMS was 31.4 μm, whereas that of 20HMS increased to 38.4 μm.

**Figure 6:**
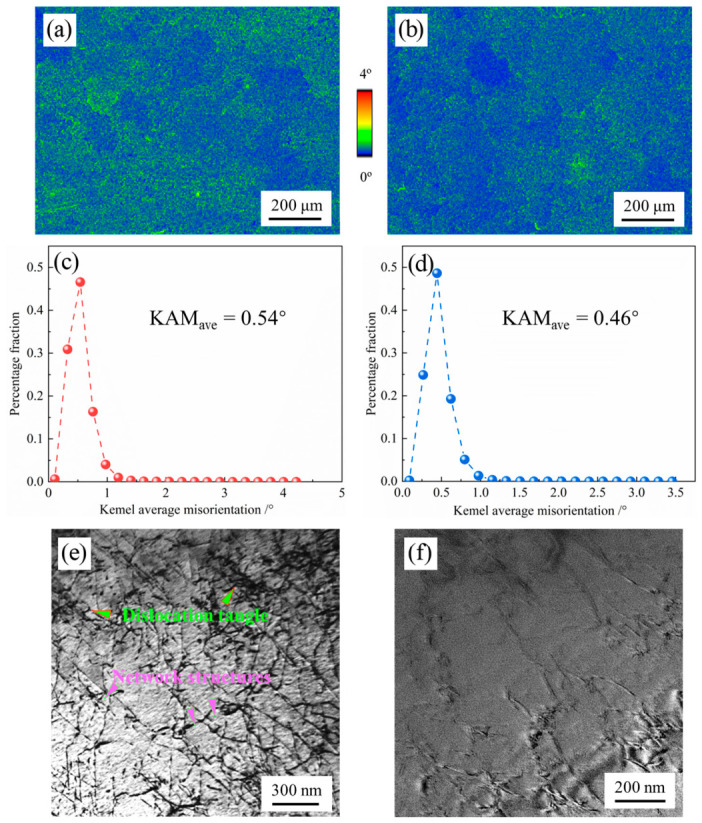
KAM maps and corresponding KAM value distributions of 15HMS (**a**,**c**) and 20HMS (**b**,**d**). TEM images of 15HMS (**e**) and 20HMS (**f**).

The distribution of kernel average misorientation (KAM) in the specimens after LTH is presented in Figure 6a,b. This result provides insight into the evolution of dislocation structures during the LTH treatment. KAM mapping has been demonstrated to be an effective method for characterizing the local strain distribution and variations in dislocation density [28]. Through statistical analysis of the corresponding KAM mapping results, KAM values under different processing conditions can be obtained quantitatively, which allows further comparison of the differences in dislocation evolution behavior. As shown in Figure 6c,d, the KAM value of 15HMS is 0.54°, which is slightly higher than that of 20HMS. This indicated a higher density of geometrically necessary dislocations (GNDs) and more severe lattice distortion in 15HMS. An elevated KAM value is associated with increased accumulation of dislocations in the vicinity of grain boundaries and sub-grain boundaries, making the slip process more easily hindered by boundary barriers and thereby shortening the effective slip distance of dislocations [29]. TEM micrographs of 15HMS showed a high density of dislocations, characterized by pronounced dislocation tangles and network structures (Figure 6e). These dislocations intersected and interacted with each other, indicating intensive dislocation multiplication and strong strain accumulation [30]. The formation of such dense dislocation configurations contributed to significant lattice distortion, which was consistent with the higher KAM values observed in the EBSD analysis (Figure 6c). By contrast, the dislocations in Figure 6f were mainly present as relatively straight and isolated segments with limited entanglement. The relatively weak interaction among dislocations prevents the formation of dense dislocation networks, as reflected by the low KAM value of 0.46° in 20HMS (Figure 6d).

### 3.2. Microhardness

**Figure 7:**
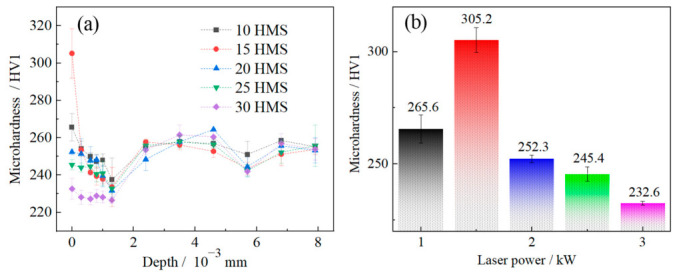
(**a**) Variation in microhardness with depth in HMS at different laser powers. (**b**) Surface microhardness of specimens after LTH treatment.

Figure 7 presents the microhardness profiles along the depth direction of specimens treated with different laser powers. As depicted, the microhardness of all specimens decreased from the top surface toward the interior, subsequently increased, and eventually stabilized at the substrate microhardness. The distribution characteristic resulted from microstructural transformations driven by the temperature gradient generated during laser surface treatment. Measurements of surface microhardness under different laser powers show that 15HMS achieved the highest value of 305.2 HV1 and displayed a pronounced strengthening effect. In contrast, the surface microhardness values of 20HMS, 25HMS, and 30HMS are all lower than that of the untreated material.

**Figure 8:**
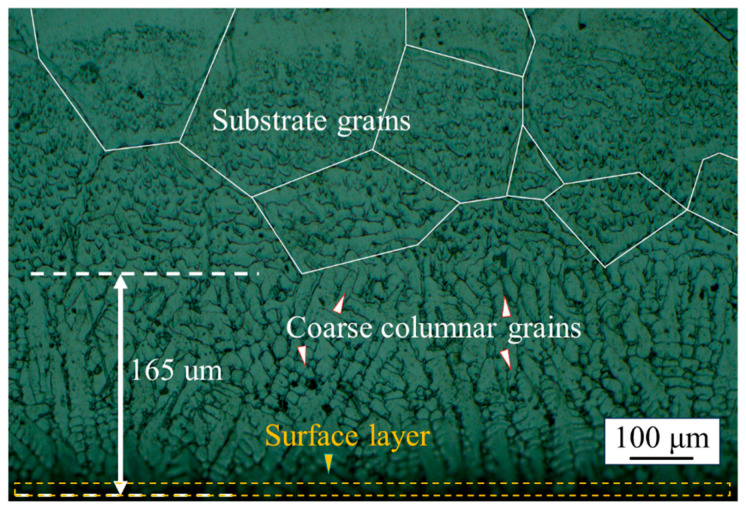
Cross-sectional metallographic image of 15HMS.

Notably, the microhardness of 15HMS reached its lowest level at a depth of approximately 165 μm. The cross-sectional OM micrograph of 15HMS is shown in Figure 8, which reveals a distinct microstructural layering around this depth. Prior to this depth, the microstructure was dominated by relatively coarse columnar grains with noticeably larger sizes, indicating a certain degree of microstructural coarsening that resulted in a progressive decrease in microhardness. The presence of coarse columnar grains at intermediate depths suggests that localized melting and resolidification may have occurred during laser processing at higher energy inputs, rather than purely solid-state transformation. Beyond approximately 165 μm, however, the columnar morphology was gradually substituted by fine equiaxed grains, and grain refinement occurred, which corresponded to the observed rebound in microhardness. As the depth further increased, the microstructure gradually transitioned into a uniform morphology that approached that of the substrate, and the corresponding microhardness also converged to the substrate level. Observations indicate that 15HMS possessed a distinct microstructural gradient along the thickness direction, and its microhardness distribution exhibited a good correlation with the variation in grain size. This gradient microstructure leads to a pronounced increase in surface microhardness, which is far superior to the marginal hardening effect (~2.56%) of the heterogeneous columnar-equiaxed dendritic structure produced by laser remelting [31].

In summary, the superior surface microhardness of 15HMS (305.2 HV1) can be attributed to a synergistic combination of multiple strengthening mechanisms, each directly supported by the experimental results presented in this study. First, grain refinement strengthening plays a key role: although the recrystallized region in 15HMS occupies a relatively large area (~87.2%), the recrystallized grains themselves remain remarkably fine (average grain size of 31.4 μm, as shown in Figure 5) due to the rapid heating and cooling cycles during laser processing combined with a relatively low peak temperature. According to the Hall-Petch relationship, a finer grain size increases the density of grain boundaries, which effectively impedes dislocation motion and thereby enhances microhardness. Second, dislocation strengthening further contributes to the observed hardening. The rapid thermal cycling and associated thermal stresses induce a high density of dislocations within the laser-treated layer, as evidenced by TEM observations (Figure 6), which reveal pronounced dislocation tangles and network structures in the 15HMS specimen. The KAM analysis supports this finding, with a KAM value of 0.54° for 15HMS, which is higher than that of 20HMS (0.46°). According to the Taylor hardening model, a higher dislocation density directly increases flow stress by restricting dislocation glide. Third, martensite-induced strengthening provides an additional but important contribution. Although the measured martensite fraction is only 3.75% (Figure 2), this small amount of hard BCC α’-martensite contributes through two path-ways: the martensite laths act as local physical barriers that impede dislocation motion, while the volume expansion associated with the martensitic transformation induces transformation-induced lattice strains and generates geometrically necessary dislocations in the adjacent austenite regions, further increasing the overall dislocation density. Therefore, while the martensite fraction is small, its contribution to strengthening is not negligible, as it synergistically interacts with the dislocation strengthening and grain refinement mechanisms. In summary, the dominant strengthening mechanisms in 15HMS are grain refinement strengthening and dislocation strengthening, with martensite-induced strengthening playing an additional but important indirect role. For 20HMS, however, the higher heat input leads to more pronounced microstructural recovery, which results in a decline in dislocation density and a coarser grain size, thereby inhibiting the formation of strengthening phases and leading to a reduction in surface microhardness.

### 3.3. Charpy Impact Performance

It is widely recognized that conventional surface-hardening techniques often de-grade impact toughness, as the formation of a hardened surface layer inevitably induces plastic deformation and residual stress concentration that compromise fracture resistance under dynamic loading. For instance, Liu et al. [25] performed high-power and high-speed laser transformation hardening on 65Mn steel and reported a 42% increase in microhardness in the laser-hardened zone, along with a significant reduction in wear depth. However, their study focused predominantly on microhardness enhancement and wear resistance, without providing quantitative data on the evolution of impact toughness following LTH treatment. Similarly, Zhao et al. [31] observed that laser remelting of Mn13 steel produced heterogeneous microstructures comprising columnar and equiaxed dendritic structures, with surface microhardness showing significant improvement under subsequent ultrasonic rolling. Yet, again, the Charpy impact toughness of the laser-remelted HMS was not quantitatively evaluated.

**Figure 9:**
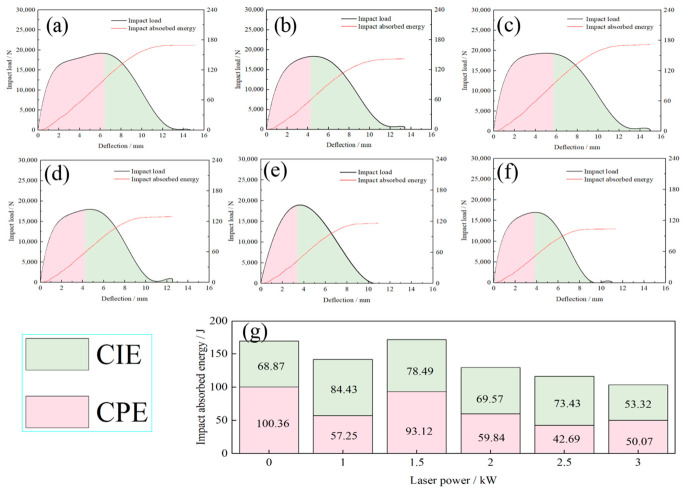
Impact load and impact absorbed energy vs. deflection curves of different laser-hardened HMSs: (**a**) Untreated HMS, (**b**) 10HMS, (**c**) 15HMS, (**d**) 20HMS, (**e**) 25HMS and (**f**) 30HMS. (**g**) Variation in CIE and CPE as a function of laser power.

To evaluate the Charpy impact performance of HMS under various laser power conditions, an oscilloscope-based impact testing system was used to capture the dynamic energy absorption behavior during the fracture process. The instantaneous relationship between load and deflection during impact was recorded, and the load-deflection data were integrated to obtain the impact load-deflection curves and the corresponding impact absorbed energy-deflection curves for the different laser power level, as shown in Figure 9.

The impact load-deflection curve can be separated by the point corresponding to the peak load into two distinct regions. The left region, highlighted in pink, is defined as the crack initiation energy (CIE), which represents the material’s the intrinsic ability l to resist crack initiation under impact loading [32]. Before the load reaches its maximum value, the energy dissipated by the applied load is generally accepted to originate primarily from the elastic and plastic deformation of the material. Crack initiation usually occurs before the peak load value is reached, as internal damage progressively accumulates with increasing load and eventually triggers the onset of cracking. Beyond the peak load, the material enters the stage of unstable crack propagation. The region on the right side of the curve, marked in green, represents the crack propagation energy (CPE). This parameter corresponds to the material’s resistance to further crack propagation after crack initiation and reflects the continuity of load-bearing and energy dissipation during impact fracture [33].

Figure 9g shows the variation in CIE and CPE for samples processed under various laser powers. The untreated HMS displayed high CIE and CPE values and reached a total impact absorbed energy (IAE) of 169.23 J, which indicates its superior overall fracture resistance. For 15HMS, the total IAE was 171.61 J and remained comparable to that of the untreated HMS. This indicated that the impact toughness was well retained after laser processing without noticeable deterioration. Contrary to the conventional view that strengthening treatments like laser transformation hardening enhance microhardness at the expense of impact toughness [34], 15HMS showed a deviation from this trend. This can be attributed to a mechanically compatible transition between the hardened layer and the substrate under the given laser power, which promotes efficient energy dissipation during impact deformation.

**Figure 10:**
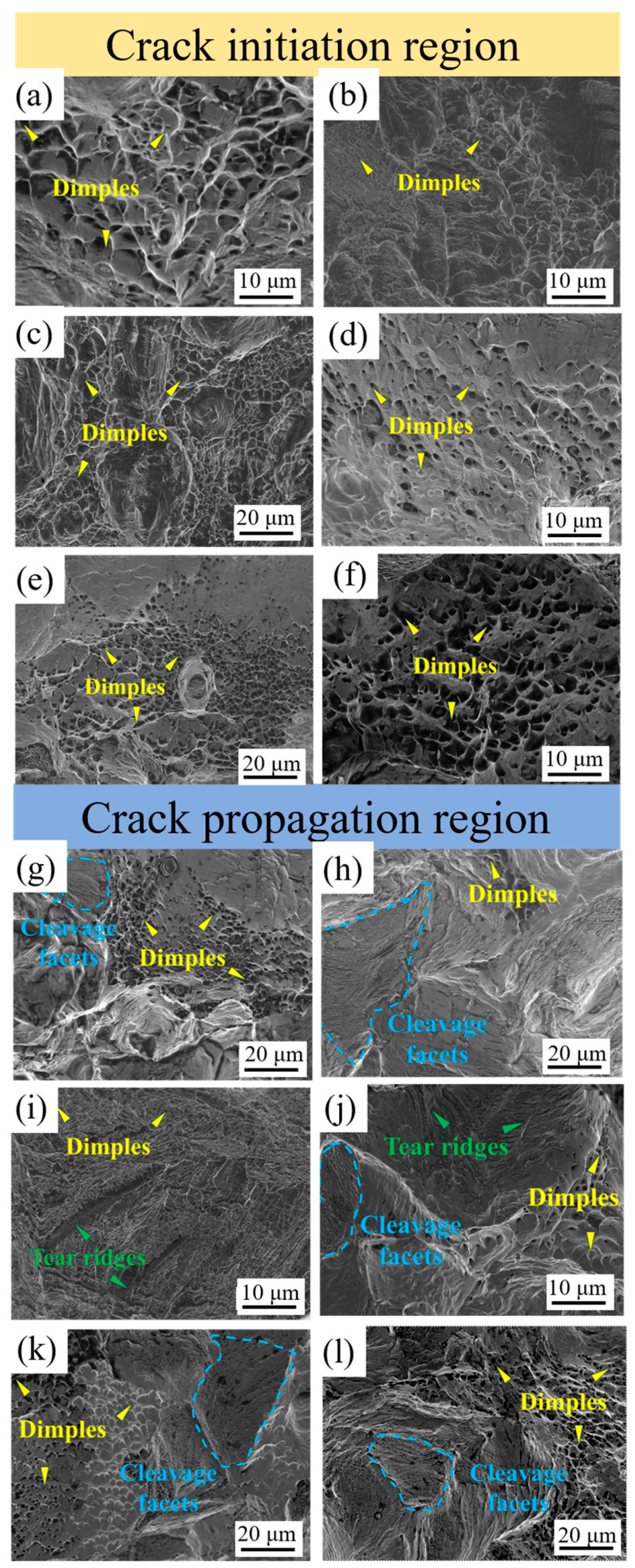
SEM images of the impact fracture surface of different laser-hardened HMSs: (**a**,**g**) Untreated HMS, (**b**,**h**) 10HMS, (**c**,**i**) 15HMS, (**d**,**j**) 20HMS, (**e**,**k**) 25HMS, and (**f**,**l**) 30HMS.

To further understand the fracture mechanisms of HMS under different laser powers, the fracture morphologies of HMS specimens were examined by SEM. Figure 10 shows the fracture surfaces of both untreated and laser-quenched HMS. It can be seen that the crack initiation regions (Figure 10a–f) in all HMSs exhibit typical ductile characteristics, manifested by the formation of voids and dimples. In comparison, the crack propagation regions (Figure 10g–l) presented diverse fracture morphologies. The differences were closely related to the changes in laser power, indicating that the employed processing conditions significantly influence the evolution of fracture behavior. As shown in Figure 10, the untreated HMS displayed dimples, small-area irregular cleavage facets and shear features, which reflected extensive plastic deformation and localized crack propagation dominated by shear mechanisms. The crack propagation region in 15HMS resembled that of the untreated HMS, both showing typical quasi-cleavage features including dimples and irregular tear ridges. This behavior corresponds to high impact energy absorption, mainly dissipated through the nucleation and coalescence of micro-voids.

### 3.4. Wear Performances

**Figure 11:**
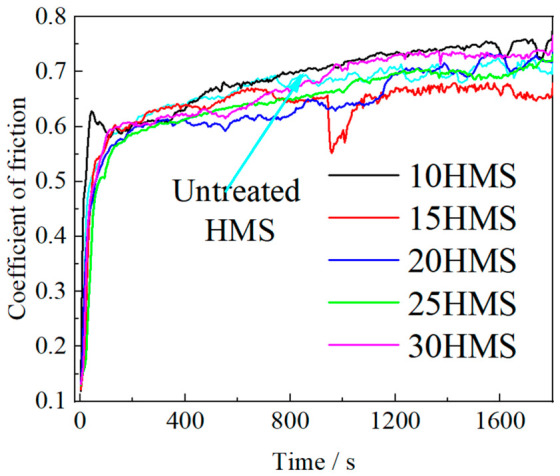
COF values of different laser-hardened HMSs.

As a key parameter, the coefficient of friction (COF) determines the functional performance of heavy-duty engineering equipment. Its stability is strongly influenced by microstructure, surface morphology, material microhardness and roughness of the worn surface [35]. Figure 11 shows the COF curves for untreated HMS and those treated with different laser powers. As can be seen, the lowest average COF value was observed for 15 HMS. According to the Archard equation, an increase in microhardness of metallic materials generally leads to a reduction in the COF, consistent with the observations in Figure 7. Laser-hardened HMSs demonstrated lower average COF values during sliding, accompanied by reduced friction at the contact interface and shear stress. This decrease in frictional force limits material removal and energy dissipation at the surface, leading to improved wear resistance of the material.

**Figure 12:**
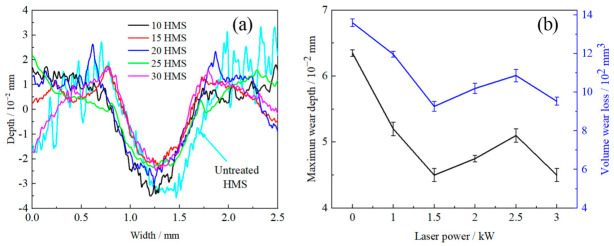
(**a**) Wear profiles of the laser transformation hardening at different powers. (**b**) Correlation of maximum wear depth and wear volume with laser transformation hardening power.

HMSs processed under dissimilar laser powers are illustrated in Figure 12a with their two-dimensional wear track profiles. It can be observed that the width of the wear tracks exhibits only minor variations. This behavior was primarily determined by the contact conditions of the friction pair, such as the applied load and the geometrical dimensions (e.g., radius) of the contact ball, which constrained the lateral expansion of the tracks under identical testing conditions. Wear depth, however, shows significant variation with laser power. It is governed by a combination of factors including the microhardness of surface, the presence of strengthening phases, and microstructural stability. LTH treatment significantly enhances the surface microhardness of HMSs and causes pronounced differences in their resistance to external load penetration and plastic deformation under different laser power conditions. Figure 12b compares the maximum wear depth and wear volume between untreated HMS and laser-hardened HMSs. Among all test conditions, 15HMS showed the lowest maximum wear depth, and its wear volume was also markedly lower than that of the other HMSs. Therefore, based on both maximum wear depth and wear volume, 15HMS demonstrated the best overall wear resistance.

**Figure 13:**
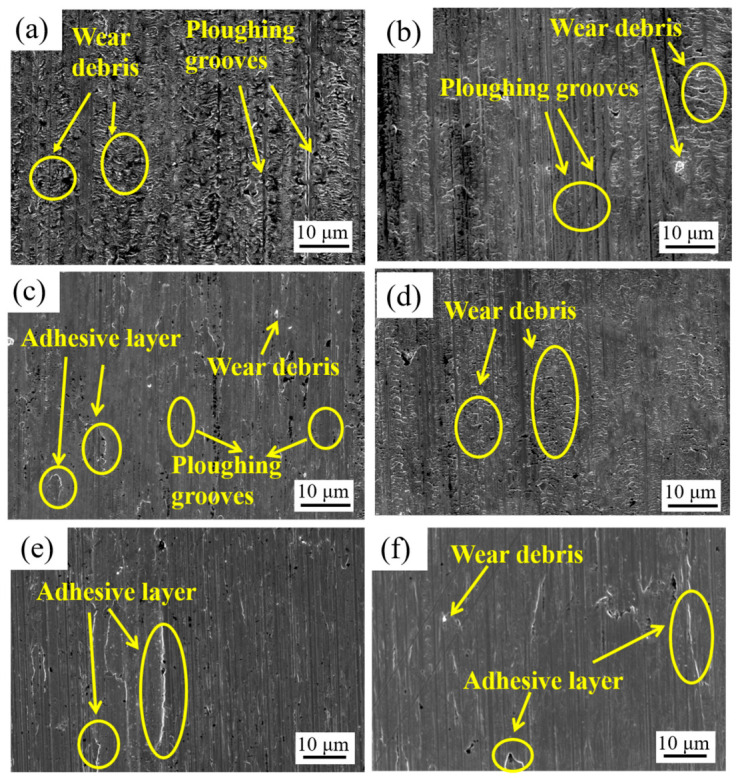
SEM images showing the surface morphologies of laser-hardened HMS after friction testing: (**a**) Untreated HMS, (**b**) 10HMS, (**c**) 15HMS, (**d**) 20HMS, (**e**) 25HMS, and (**f**) 30HMS.

To directly compare the influence of various laser power treatments on the worn surface morphology of HMS, the worn surfaces of HMS after testing were systematically observed and characterized using SEM. Figure 13 presents the worn surface morphologies of laser-hardened HMS. The untreated HMS exhibited multiple wear features, and the surface damage was relatively severe. Distinct grooves along the sliding direction were observed, with material squeezed out on both sides due to softening, forming pronounced ridges. Large-area spalling regions suggest the delamination of the surface layer, which is mainly attributed to the propagation of subsurface cracks and adhesive fatigue. As the laser power increased, the worn surfaces gradually became smoother, and the adhesive layers became sparse and discontinuous. Only a small number of fragmented debris remained on the surface. Clear debris accumulation and groove morphology remained on the worn surfaces of 10HMS and 20HMS, and these features indicated the dominance of adhesive wear. For 15HMS, 25HMS and 30HMS, little or no debris appeared on the worn surfaces, and the surfaces mainly displayed a single groove morphology. The dominant wear mechanism therefore changed gradually from adhesive wear to abrasive wear. Under the same applied load, 10HMS showed greater debris accumulation, wider grooves, and more pronounced spalling compared with 20HMS. This demonstrated that 10HMS suffered a higher degree of surface damage and exhibited inferior wear resistance. Overall, the increase in laser power markedly modifies the wear morphology of HMS. The reduction in debris accumulation, combined with more pronounced grooves and surface spalling, reflects a transition in the wear mechanism from accumulation-dominated to scratch- and spalling-dominated wear.

**Figure 14:**
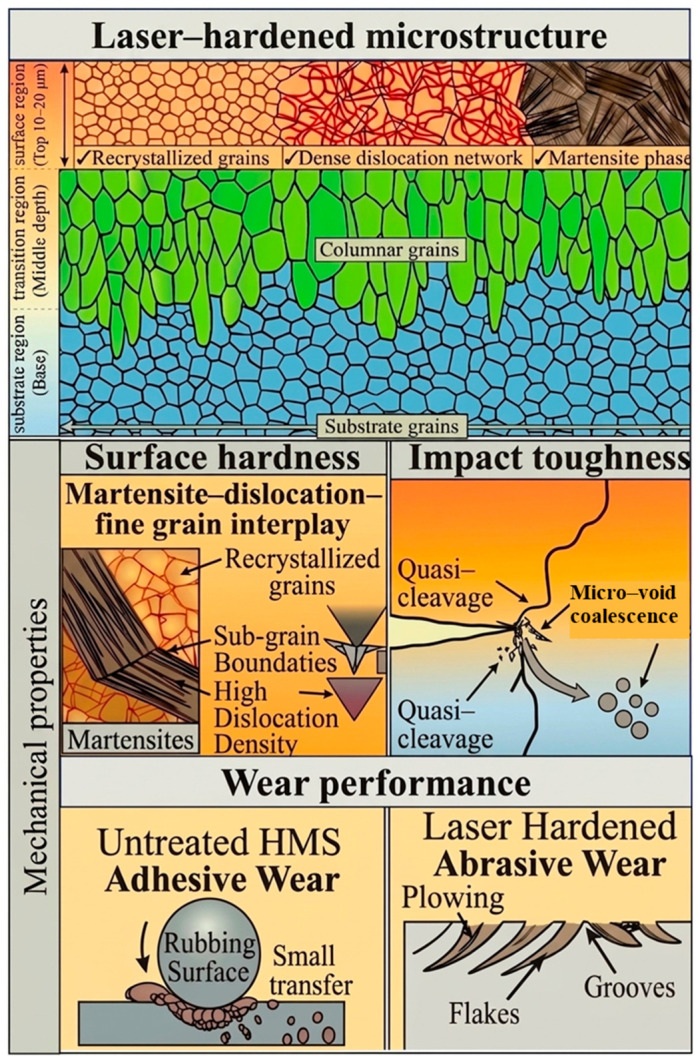
Schematic diagram illustrating the microstructural evolution and mechanical properties changes in HMS after laser transformation hardening.

To conclude the findings discussed above, a mechanism diagram in Figure 14 has been constructed to visually summarize the key insights derived from the experimental results and analyses. The diagram illustrates the correlation between laser power treatment, microstructure evolution, and the resulting mechanical properties, particularly the phase transformation, dislocation density, microhardness distribution, fracture behavior, and wear performance.

Despite the promising results demonstrated above, several limitations of the present work should be acknowledged to guide future research. First, only laser power was varied while the scanning speed remained constant; the combined effects of other parameters (e.g., scanning speed, spot size) on the temperature gradient and micro-structural evolution warrant further investigation. Second, the wear tests were con-ducted under reciprocating sliding conditions, which may not fully replicate the complex impact-abrasion environments encountered in actual service applications (e.g., mining machinery), and the long-term stability of the laser-quenched layer remains to be evaluated.

## 4. Conclusions

This work investigated surface strengthening of M13 high-manganese steel via laser transformation hardening. The effects of laser power on microstructural evolution, including grain refinement and phase transformation, were systematically examined. These microstructural changes were correlated with mechanical properties such as microhardness, impact toughness, and wear resistance. The key conclusions drawn from this study are summarized as follows:

(1) Laser transformation hardening at 1.5 kW produced a well-defined gradient microstructure. A non-monotonic microhardness profile was observed in laser-quenched HMS, arising from a columnar-to-equiaxed transition: coarse columnar grains at intermediate depths caused a slight microhardness decrease, while grain refinement near the substrate led to microhardness recovery. This confirms that exploiting the temperature gradient enables tailored depth-dependent microhardness distribution. Laser transformation hardening.
(2) The surface layer at 1.5 kW exhibited fine recrystallized grains, a dense dislocation network, and a small fraction of martensite. Incomplete dynamic recrystallization contributed to dislocation accumulation, while grain refinement impeded dislocation motion, and the martensitic phase provided additional resistance to plastic deformation. These features collectively increased surface microhardness by 1.3-fold compared to untreated HMS. Laser transformation hardening.
(3) Despite significant surface hardening, the laser-quenched specimen retained high impact toughness of 171.61 J. The gradient microstructure and mechanical compatibility enabled coordinated deformation, with quasi-cleavage fracture facilitating energy dissipation through micro-void nucleation. Thus, the toughness remained comparable to that of the untreated material, demonstrating that the gradient design avoids the typical microhardness-toughness trade-off.
(4) The combination of surface hardening and microstructural refinement limited frictional damage and material removal. Incomplete recrystallization governs a gradual transition of the dominant wear mechanism from adhesion-driven debris accumulation to abrasion-dominated grooves and spalling. This finding moves beyond parameter optimization by establishing a microstructure-based criterion for wear resistance enhancement.

## Data Availability

The original contributions presented in this study are included in the article. Further inquiries can be directed to the corresponding author.
